# Gatifloxacin Induces S and G_2_-Phase Cell Cycle Arrest in Pancreatic Cancer Cells via p21/p27/p53

**DOI:** 10.1371/journal.pone.0047796

**Published:** 2012-10-25

**Authors:** Vikas Yadav, Sarwat Sultana, Jyoti Yadav, Neeru Saini

**Affiliations:** 1 Institute of Genomics and Integrative Biology (CSIR), Mall Road, Delhi, India; 2 Department of Medical Elementology and Toxicology, Jamia Hamdard (Hamdard University), Hamdard Nagar, Delhi, India; Technische Universität München, Germany

## Abstract

Pancreatic cancer, despite being the most dreadful among gastrointestinal cancers, is poorly diagnosed, and further, the situation has been aggravated owing to acquired drug resistance against the single known drug therapy. While previous studies have highlighted the growth inhibitory effects of older generation fluoroquinolones, the current study aims to evaluate the growth inhibitory effects of newer generation fluoroquinolone, Gatifloxacin, on pancreatic cancer cell lines MIA PaCa-2 and Panc-1 as well as to elucidate the underlying molecular mechanisms. Herein, we report that Gatifloxacin suppresses the proliferation of MIA PaCa-2 and Panc-1 cells by causing S and G_2_-phase cell cycle arrest without induction of apoptosis. Blockade in S-phase of the cell cycle was associated with increased TGF-β1 expression and translocation of Smad3-4 complex to the nucleus with subsequent activation of p21 in MIA PaCa-2 cells, whereas TGF-β signalling attenuated Panc-1 cells showed S-phase arrest by direct activation of p27. However, Gatifloxacin mediated G_2_–phase cell cycle arrest was found to be p53 dependent in both the cell lines. Our study is of interest because fluoroquinolones have the ability to penetrate pancreatic tissue which can be very effective in combating pancreatic cancers that are usually associated with loss or downregulation of CDK inhibitors p21/p27 as well as mutational inactivation of p53. Additionally, Gatifloxacin was also found to synergize the effect of Gemcitabine, the only known drug against pancreatic cancer, as well as the broad spectrum anticancer drug cisplatin. Taken together our results suggest that Gatifloxacin possesses anticancer activities against pancreatic cancer and is a promising candidate to be repositioned from broad spectrum antibiotics to anticancer agent.

## Introduction

Pancreatic cancer, the malignant neoplasm of the pancreas is currently the fifth most common cause of cancer death [1]. Despite improvements in diagnosis, the prognosis still remains poor because of delayed symptom presentation, aggressive tumor growth and profound desmoplastic reaction [2]; [3]. The 5-year survival rate is approximately 15–20%, following pancreas resection in case of pancreatic cancer [4]. Apart from surgery, adjuvant chemotherapy with Gemcitabine and erlotinib has been shown to improve prognosis in resectable pancreatic cancer cases [5]. So, there is an increase in survival rate with conventional cytotoxic chemotherapy as compared to surgery [6], however still there’s need to develop or identify the potential anticancer drug with increased selectivity and reduced toxicity.

In recent years older generation fluoroquinolones such as Moxifloxacin and Ciprofloxacin possessing broad spectrum antibiotic activity, have shown growth inhibitory effects by inducing apoptosis and cell cycle arrest in various cancer cell lines [7]–[10]. These nonantimicrobial activities have rendered them unique among other broad spectrum antibiotics. Gatifloxacin or 8-methoxy fluoroquinolones is the newer (fourth) generation fluoroquinolone which shows similar antibiotic effects by targeting bacterial DNA gyrase and Topoisomerase [11]–[13], and is also a potent drug in several highly infectious diseases such as, the Sexually Transmitted Diseases, Toxoplasmosis and Tularaemia [14]–[16]. Like other fluoroquinolone family members it is also known to have certain immunomodulatory effects *in vitro* in various cell lines as well as it is under clinical trials for treatment of pulmonary tuberculosis [17]; [18]. Having so much similar implications with other fluoroquinolones family members, no growth inhibitory activity against cancer cell lines has been reported for Gatifloxacin. Moreover, the pancreatic tissue penetrating efficiency is very well reported [19] in case of fluoroquinolones which tempted us to explore the mechanism by which Gatifloxacin suppresses pancreatic cancer cell growth. In this study, we investigated the effect of Gatifloxacin on survival and proliferation of pancreatic cancer cell lines (MIA PaCa-2 and Panc-1) and found that Gatifloxacin was able to suppress proliferation of both cell lines, with MIA PaCa-2 being more sensitive than Panc-1. The differential outcome might be because of difference in functional TGF-β receptors in the two cell lines with MIA PaCa-2 being sensitive to TGF-β1 and Panc-1 being resistant as they lack functional TGF-β type I receptor [20]. We found that Gatifloxacin arrests cells in G_2_-phase via inactivation of cdc2-cyclinB1 complex by phosphorylation of cdc2 on Tyr15 through p53 activation. Moreover we show that Gatifloxacin can induce p21 and p27 levels in MIA PaCa-2 and Panc-1 cells respectively thereby causing S-phase arrest. We further demonstrated that Gatifloxacin was able to synergize the antiproliferative activity of Gemcitabine and Cisplatin in both cell lines.

## Results

### Gatifloxacin Suppresses Proliferation of Pancreatic Carcinoma Cells *In Vitro*

**Figure 1:**
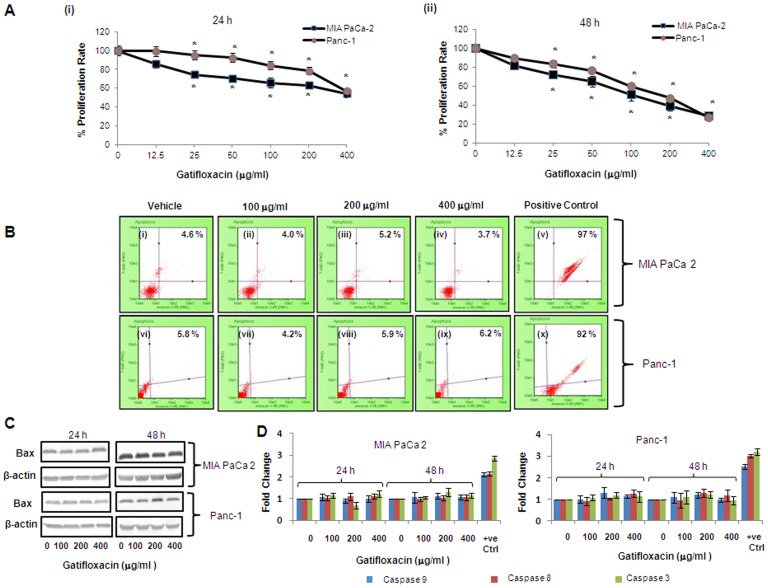
Gatifloxacin inhibits proliferation of cultured pancreatic cancer cells without induction of Apoptosis. MTT assay of MIA PaCa-2 and Panc-1 cells after treatment with Gatifloxacin (0–400 µg/ml) for 24 h (**Ai**) and 48 h (**Aii**)**.** Cells were seeded in 96 well plates (1×10^4^ cells/well) which were allowed to adhere overnight and were subsequently treated with increasing concentration of Gatifloxacin for 24 and 48 h. Vertical axis represents % proliferation rate whereas Horizontal axis represents increasing concentration of Gatifloxacin in µg/ml. Data are mean ± SEM three independent experiments performed in triplicate. * p<0.01 compared to vehicle control. (**B**) Annexin V-PE binding in MIA PaCa-2 (i-v) and Panc-1 (vi-x) after treatment with Gatifloxacin for 48 h as evaluated by 7-AAD and AnnexinV staining. (i) and (vi) Vehicle treated control cells, (ii) and (vii) cells treated with 100 µg/ml, (iii) and (viii) cells treated with 200 µg/ml, (iv) and (ix) cells treated with 400 µg/ml of Gatifloxacin. (v) MIA PaCa-2 cells and (x) Panc-1 cells treated with curcumin 60 µM for 24 h as positive control. Vertical axis represents 7-AAD positive cells whereas horizontal axis represents Annexin V-PE positive cells. Representative of three independent experiments has been shown with similar results. (**C**) Western blot analysis of the expression of Bax protein under the effect of Gatifloxacin in a time (24, 48 h) and dose (0, 100, 200, 400 µg/ml) dependent manner. β-Actin was used as a loading control. (**D**) Caspase 3, 8, 9 activity of MIA PaCa-2 and Panc-1 cells treated with Gatifloxacin in time (24, 48 h) and dose (0, 100, 200, 400 µg/ml) dependent manner. Bar graph represents mean ± SEM from three independent experiments.

To evaluate the anti-proliferative effect of Gatifloxacin, we used MIA PaCa-2 and Panc-1 pancreatic carcinoma cell lines. We first studied the effect of different doses (0–400 µg/ml) of Gatifloxacin on the viability of MIA PaCa-2 and Panc-1 cells for 24 h and 48 h using MTT assay. As shown in Figure 1, GFX treatment resulted in time and dose-dependent decrease in cell proliferation in MIA PaCa-2 and Panc-1 cells albeit at different levels. Gatifloxacin treatment resulted in 15–46% (p = 0.002) decrease in cell viability in MIA PaCa-2 and 1–43% (p = 0.007) decrease in cell viability in Panc-1 cells after 24 h respectively (Figure 1A i). We observed 19–73% (p = 0.0016) decrease in cell viability in MIA PaCa-2 and 11–72% (p = 0.00016) decrease in cell viability in Panc-1 cells at 48 h respectively (Figure 1A ii). The above data clearly shows MIA PaCa-2 to be more sensitive to Gatifloxacin than Panc-1 at all doses. A striking observation from this data was the decrease in viability, being more pronounced at higher doses (100, 200 and 400 µg/ml) of Gatifloxacin treatment after 24 h and 48 h treatment in both cell lines and hence we took these concentrations and time points to carry out further experiments.

### Gatifloxacin Induces Cell Cycle Arrest at S and G_2_ - Phase in Pancreatic Carcinoma Cells without Induction of Apoptosis

**Figure 2:**
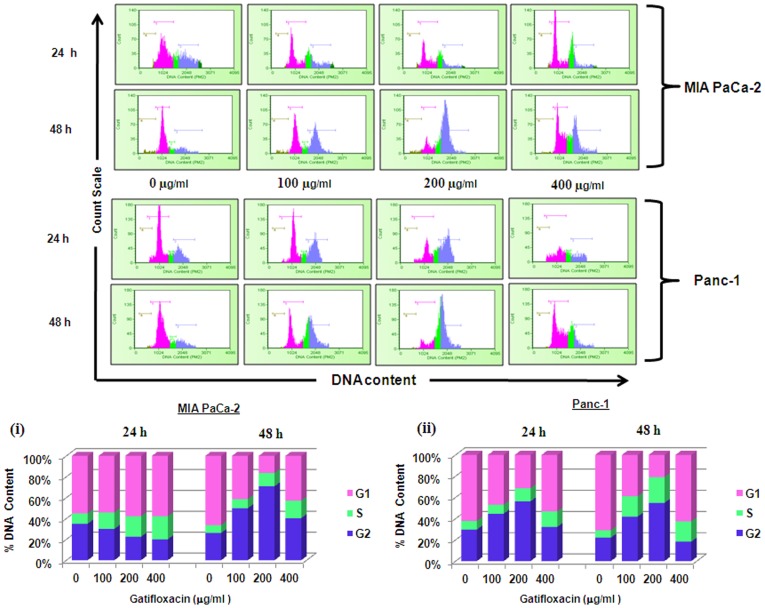
Gatifloxacin induces S and G_2_ phase cell cycle arrest in pancreatic cancer cells. Effects of Gatifloxacin on cell cycle were investigated using PI (Propidium Iodide) staining. Cells were treated with (0–400 µg/ml) Gatifloxacin for 24 and 48 h, collected and stained with PI. Here Pink peak represents G_1_-phase, Green peak represents S-phase and Blue peak represents G_2_-phase respectively. Upper panel shows representative of three independent experiments with similar results and lower panel represents the bar diagram of cells in different phases. Bar graph represents mean ± SEM from three independent experiments. (**i**) Representative bar graph for MIA PaCa-2, (**ii**) Representative bar graph for Panc-1.

We next investigated whether Gatifloxacin-mediated decrease in viability of MIA PaCa-2 and Panc-1 cells is because of apoptosis/necrosis. We first checked for the induction of apoptosis by annexin assay. As shown in Figure 1B we didn’t find any significant changes in apoptotic/necrotic population at all the doses of Gatifloxacin as compared to vehicle treated cells in both cell lines at 24 h as well as at 48 h respectively. To further cross validate our annexin data, we next checked the expression level of proapoptotic protein Bax by western blotting and activity of caspase -3, -8 and -9 in a dose and time dependent manner under the effect of Gatifloxacin. We did not find any significant change either in Bax protein level (Figure 1C) or caspase -3, -8 and -9 activity (Figure 1D), which shows that Gatifloxacin does not hinder the viability of cells. Results of Annexin V and Caspase activity were also validated using a positive control, curcumin (60 µM for 24 h). *We next* measured the *cell cycle* status of MIA PaCa-2 and Panc-1 cells in presence of 100, 200, 400 µg/ml of Gatifloxacin at 24h and 48h respectively (Figure 2). Cell cycle distribution analysis showed that Gatifloxacin hampers the cell cycle progression by arresting the cells in S and G_2_-phase in both the cell lines. In MIA PaCa-2 cells, increase in the percentage of cells in S-phase was from 9±1.1% (vehicle treated cells) to 21±2.1% (400 µg/ml), with concomitant decrease in percentage of cells in G_2_ phase from vehicle treated 31±2.1% to 18±2.7% (400 µg/ml) and no change in the percentage of cells in G_1_ phase was observed in cells exposed till 24 h. However by 48 hrs, cells started accumulating in G_2_ phase from 24±3% (vehicle treated) to 67±2.5% at 200 µg/ml of Gatifloxacin and this was reduced to 40±2% at 400 µg/ml of Gatifloxacin (Figure 2i and upper panel). In Panc-1, cells started accumulating in S-phase from 8±1% (vehicle treated) to 14±1.5% (400 µg/ml), but unlike MIA PaCa-2, cells started accumulating in G_2_-phase from 24 h onwards from 29±1% (vehicle treated) to 56±3% and was restricted back to 32±1.05% at 400 µg/ml (Figure 2ii and upper panel). No increase in SubG1 peak was observed in both the cell lines. Taken together our data strongly suggest that Gatifloxacin does not induce apoptosis but causes an arrest of cells in S and G_2_-phase in pancreatic carcinoma cells.

### Gatifloxacin causes Activation of TGF-β1/SMAD Complex/p21 in MIA PaCa-2 and Activation of p27 in Panc-1

**Figure 3:**
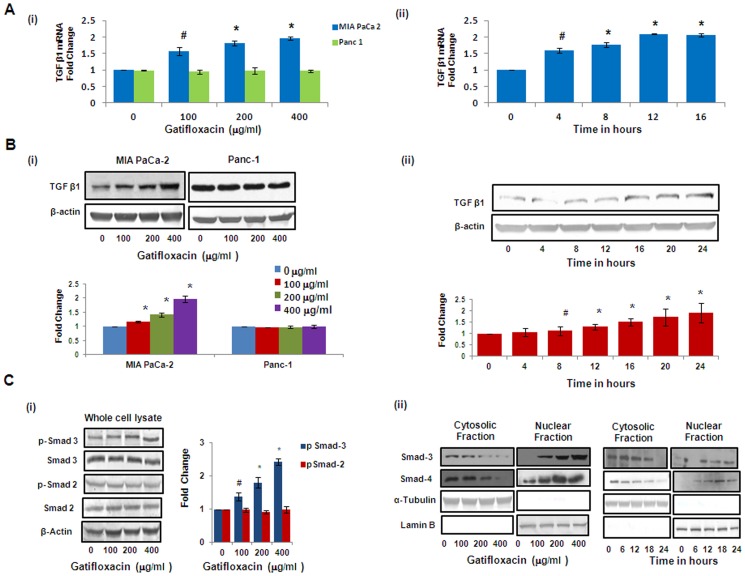
Gatifloxacin causes activation of TGF-β1 and Smad Complex in MIA PaCa-2. (**A**) (**i**) Real Time PCR analysis of TGF-β1 expression in MIA PaCa-2 and Panc-1 cells treated with Gatifloxacin in a dose dependent manner, (**ii**) Real Time PCR analysis of TGF-β1 expression in MIA PaCa-2 cells treated with 400 µg/ml of Gatifloxacin in a time dependent manner. 18S rRNA was used to normalize the results. (**B**) Western blot analysis of TGF-β1 expression in MIA PaCa-2 and Panc-1 cells treated with Gatifloxacin in a dose dependent manner (**i**) western blot analysis of TGF-β1 expression in MIA PaCa-2 cells treated with 400 µg/ml of Gatifloxacin in a time dependent manner (**ii**). Data are representative of typical experiment repeated three times with similar results. Bar Graph represents the mean ± SEM. (**C**) (**i**) Effect of Gatifloxacin on receptor mediated Smads (pSmad-2 and pSmad-3) when assessed in whole cell lysate in MIA PaCa-2 cells. β-actin was used as a loading control. (**ii**) Translocation of Smad 3–4 complex from cytoplasm to nucleus under the effect of Gatifloxacin in a dose (0, 100, 200, 400 µg/ml) and time dependent manner (0, 6, 12, 18, 24 h) was assessed by western blotting. Nuclear and Cytoplasmic fractions were separated as described in the “Material and methods” section. Lamin B (Nuclear specific protein) and α-Tubulin (Cytoplasmic specific protein) were used as loading controls.

**Figure 4:**
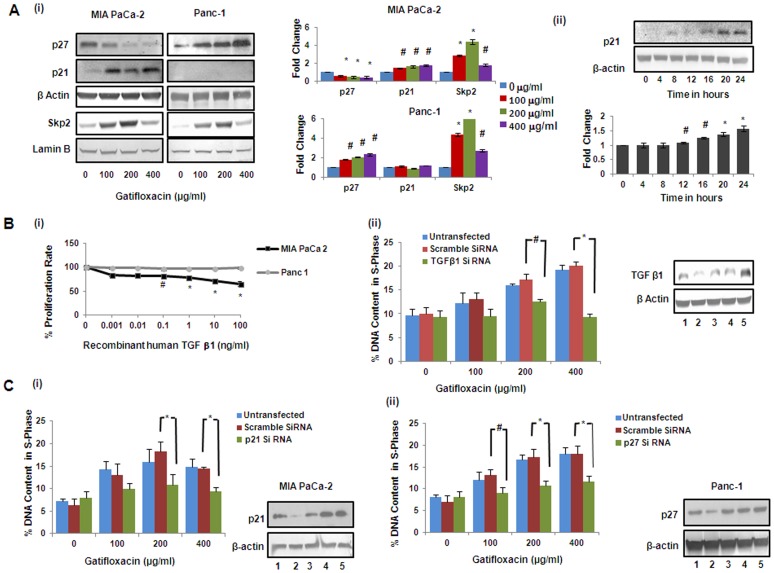
Gatifloxacin causes activation of p21 in MIA PaCa-2 and p27 in Panc-1 cells, but Skp2 in both cell lines. (**A**) (**i**) Western blot analysis of p21, p27 and Skp2 expression in MIA PaCa-2 and Panc-1 cells treated with Gatifloxacin in a dose dependent manner. β-Actin was used as loading control for p21 and p27, whereas Lamin B was used as loading control for Skp2, (**A**) (**ii**) Western blot analysis of p21 expression in MIA PaCa-2 cells treated with 400 µg/ml of Gatifloxacin in a time dependent manner. Data are representative of typical experiment repeated three times with similar results. Bar Graph represents the mean ± SEM. *p<0.01, #p<0.05 versus control. **Gatifloxacin mediated S phase arrest is TGF-β1 dependent in MIA PaCa-2** (**B**) (**i**) MTT assay of MIA PaCa-2 and Panc-1 cells in presence of recombinant-TGF-β1 for 48 h. Vertical axis represents % proliferation rate whereas Horizontal axis represents increasing concentration of recombinant-TGF-β1 in ng/ml. Data are mean ± SEM three independent experiments performed in triplicate. *p<0.01, #p<0.05 versus control. (**B**) (**ii**) Abolishment of S-phase arrest in MIA PaCa-2 cells as assessed by PI (Propidium Iodide) staining. Cells transfected with TGF-β1 siRNA (48 h) and scrambled siRNA alongwith untransfected cells were treated from 100 µg/ml to 400 µg/ml Gatifloxacin (24 h) after 24 h of transfection, collected and stained with PI. Left panel represents the bar graph where vertical axis represents %DNA content in S-phase and Horizontal axis represents Gatifloxacin concentration in µg/ml. Bar graph represents mean ± SEM from three independent experiments. *p<0.01, #p<0.05 versus control. Right panel shows western blot for the knockdown efficiency of TGF-β1 siRNA. (1) represents untransfected control cells, (2) TGF-β1 siRNA transfected cells, (3) TGF-β1 siRNA transfected cells with 400 µg/ml of Gatifloxacin, (4) siRNA transfected cells, (5) siRNA transfected cells with 400 µg/ml of Gatifloxacin. **Gatifloxacin mediated S phase arrest is p21 dependent in MIA PaCa-2 and p27 dependent in Panc-1 cells.** (**C**) Abolishment of S-phase arrest in MIA PaCa-2 cells transfected with p21 siRNA (**i**) and Panc-1 cells transfected with p27 siRNA (**ii**)**,** respectively as assessed by PI (Propidium Iodide) staining. Cells transfected with p21/p27 siRNA and scrambled siRNA (48 h) alongwith untransfected cells were treated with 100 µg/ml to 400 µg/ml of Gatifloxacin (24 h) after 24 h of transfection, collected and stained with PI. Left panel represents the bar graph where vertical axis represents %DNA content in S-phase and Horizontal axis represents Gatifloxacin concentration in µg/ml. Right panel shows western blot for the knockdown efficiency of p21 or p27 siRNA. (1) represents untransfected control cells, (2) p21/p27 siRNA transfected cells, (3) p21/p27 siRNA transfected cells treated with 400 µg/ml of Gatifloxacin, (4) scrambled siRNA transfected cells, (5) scrambled siRNA transfected cells treated with 400 µg/ml of Gatifloxacin. Bar graph represents mean ± SEM from three independent experiments. *p<0.01, #p<0.05 versus control.

Recent studies have shown that one of the fluoroquinolone, ciprofloxacin suppresses colon cancer cell proliferation by arresting them in S-phase through TGF-β1 and TGF-β1 is also very well known to cause cell cycle arrest in pancreatic cancer cells [21]; [22]. To *investigate* whether Gatifloxacin-*induced* S-phase arrest was *TGF*-β *dependent* we checked the levels of TGF-β1 at the transcriptional as well as translational levels. We found Gatifloxacin treatment (100, 200, 400 µg/ml) to increase TGF-β1 expression in a dose dependent manner by 1.5–2 fold (p<0.01) at the transcriptional level and 1.3–2 fold (p<0.01) at translational level after 48 h in MIA PaCa-2 cell but not in Panc-1 cells (Figure 3Ai, 3Bi). Since we *found significant increase in* TGF-β1 expression level at 400 µg/ml, we did the time-course study and observed that TGF-β1 started increasing transcriptionally within 4 h (p = 0.023) of treatment and reached maximum by 12 h (p<0.01) and then plateaued at 16 h (Figure 3Aii). However translationally, TGF-β1 gets activated within 8 h (p = 0.039) only and reached to its maximum expression by 16 h (p<0.01) (Figure 3Bii), as assessed by real time PCR and western blotting. There is increasing evidence that Smad transcription factors mediate the growth inhibitory effect of transforming growth factor-β1 (TGF-β1) in many cell types [23]. To investigate the relative contribution of individual Smad proteins to TGF-β1 signaling, protein expression of Smad2 and Smad3 was analysed by western blotting of cell extracts after 48 h of Gatifloxacin treatment (100, 200, 400 µg/ml) in MIA PaCa-2 cells. As shown in Figure 3Ci, we observed significant increase in phospho-Smad3 **(** *pSmad3 at Ser 423*) whereas there was no change in the levels of *phospho*-*Smad2* **(** *pSmad2 at Ser 467* **)** after 48 h of Gatifloxacin treatment at all doses in MIA PaCa-2 cells. Furthermore, the increase in phospho-*Smad3* **(** *pSmad3* **)** was found to be in a dose dependent manner (1.3–2.4 fold, p = 0.0127) as compared to vehicle treated cells. Enough evidence is there to support that activated Smad2 or Smad3 dimerizes with Smad4 and translocates to the nucleus, whereby the activated complexes associate with Smad-binding element (SBEs) in promoters of numerous genes leading to their induction [24]–[26]. p21^Waf1/Cip1^ is one such gene which is known to be involved in the TGF-β1 mediated regulation of cell proliferation [27]. To determine whether Smad3 translocates in response to TGF-β1 stimulation in MIA PaCa-2 cells, we assessed the subcellular distribution of the Smad3/4 and as expected, significant decrease in the Smad-3 and Smad-4 protein expression was observed in the cytosolic fraction and there was concomitant increase in the Smad-3 and Smad-4 protein expression in nuclear fraction in Gatifloxacin treated MIA PaCa-2 cells in a dose and time dependent manner (Figure 3Cii). Simultaneously we also analysed the expression of p21^Waf1/Cip1^, the downstream effector of TGF-β1 which is known to be a crucial regulator of cell cycle progression. Like p21, p27 is a cyclin dependent kinase inhibitor that regulates G_1_-S phase of cell cycle progression, hence we checked the expression of p21 and p27 by western blot analysis [28]. We observed significant increase (1.4–1.7 fold, p = 0.038) in the levels of p21 and significant decrease (0.57–0.35 fold, p = 0.012) in the levels of p27 in a dose dependent manner after 48 h of Gatifloxacin treatment as compared to vehicle treated cells (Figure 4Ai). Interestingly increase in p21 was observed only after TGF-β1 activation i.e after 12 h (p = 0.035), in MIA PaCa-2 cells (Figure 4Aii). However in case of Panc-1 cells that lacks functional TGF-β1 we didn’t find activation of p21, but we do find the activation of p27. We observed significant increase (1.8–2.3 fold, p = 0.021) in the levels of p27 in a dose dependent manner after 48 h of Gatifloxacin treatment as compared to vehicle treated cells (Figure 4Ai). Simultaneously we also checked the levels of Skp2 which mediates ubiquitination and degradation of p27/p21 in both the cell lines MIA PaCa-2 and Panc-1 in response to Gatifloxacin treatment (0, 100, 200 and 400 µg/ml) [29]. We found significant increase (2.7–4.3 fold, p = .008 in MIA PaCa-2 cells and 4.3–6.3 fold p = 0.005 in Panc-1) in Skp2 levels in both the cell lines. As expected we found inverse correlation between Skp2 and p27/p21 (Figure 4Ai). Taken together this study indicates that TGF-β1/Smad3/p21, is one of the pathways that leads to S-phase arrest in MIA PaCa-2 cells and p27 playing crucial role in S-phase arrest of Panc-1 cells.

### Gatifloxacin Mediated S-phase Arrest is TGF-β1/p21 Dependent in MIA PaCa-2 and p27 Dependent in Panc-1 Cells

To confirm the role of TGF-β1 in suppressing the proliferation, recombinant TGF-β1 was transfected at varied doses (0–100 ng/ml) in MIA PaCa-2 and Panc-1 cells for 48h and MTT assay was performed. We observed that over expression of TGF-β1 significantly suppressed MIA PaCa-2 cell proliferation in a dose dependent manner. We observed significant decrease (35%, p<0.05, Figure 4Bi) in cell proliferation at a concentration of 100 ng/ml in MIA PaCa-2 cells but not in Panc-1 cells. Our findings are consistent to the findings of Francisco j nicolas *et al* which suggests Panc-1 to be resistant to TGF-β1 induced growth arrest. Further to confirm the role of TGF-β1 in mediating S-phase arrest induced by Gatifloxacin in MIA PaCa-2, we silenced the TGF-β1 expression by using siRNA and did cell cycle analysis. As shown in Figure 4Bii, S-Phase arrest was totally abolished at all the three doses of Gatifloxacin used in TGF-β1 siRNA transfected cells as compared to scrambled siRNA transfected cells or untransfected cells. Accordingly, we next sought to determine the role of p21 and p27 on S-phase arrest in TGF-β1 sensitive MIA PaCa-2 or resistant Panc-1 cells respectively. We found that p21 siRNA transfected MIA PaCa-2 (Figure 4Ci) and p27 siRNA transfected Panc-1 cells (Figure 4Cii) strongly inhibits the Gatifloxacin induced S-phase arrest as compared to scrambled siRNA at all the doses. siRNA mediated knock down of TGF-β1, p21 and p27 was also confirmed using western blot analysis (Figure 4Bii, Ci and Cii). siRNA mediated knockdown results suggest that S phase arrest of Gatifloxacin is TGF-β1/p21 dependent in MIA PaCa-2 and p27 dependent in Panc-1 cells.

### Gatifloxacin Inhibits pAKT and causes G_2_–phase Arrest in p53 Dependent Manner in Both Cell Lines

**Figure 5:**
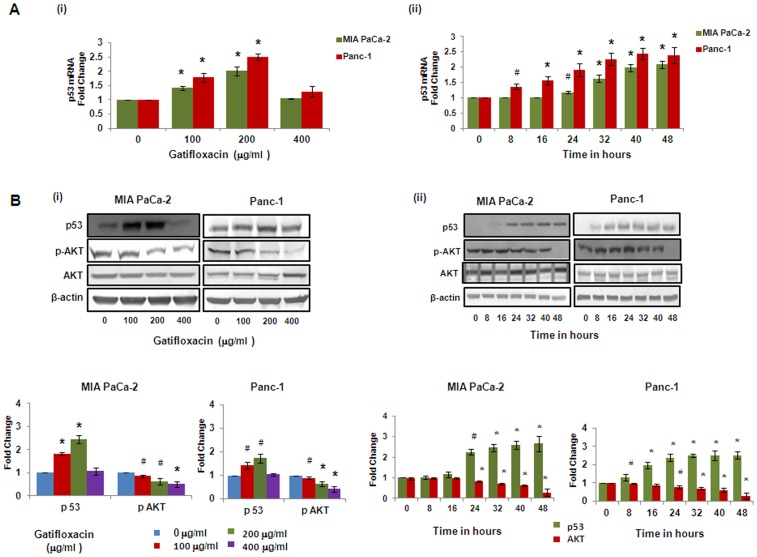
Gatifloxacin Causes activation of p53 and inhibits pAKT. (**A**) (**i**) Real Time PCR analysis of p53 expression in MIA PaCa-2 and Panc-1 cells treated with Gatifloxacin in a dose dependent manner, (**ii**) Real Time PCR analysis of p53 expression in MIA PaCa-2 and Panc-1 cells treated with 200µg/ml of Gatifloxacin in a time dependent manner. 18S rRNA was used to normalize the results. (**B**) (**i**) Western blot analysis of p53, AKT and pAKT expression in MIA PaCa-2 and Panc-1 cells treated with Gatifloxacin in a dose dependent manner, (**ii**) Western blot analysis of p53, AKT and pAKT expression in MIA PaCa-2 and Panc-1 cells treated with Gatifloxacin in a time dependent manner. β-actin was used as loading control. Data are representative of typical experiment repeated three times with similar results. Bar Graph represents the mean ± SEM. *p<0.01, #p<0.05 compared to control.

Various studies have shown that p53 can activate the expression of the p21 which then inhibits cyclin dependent kinases and leads to cell cycle arrest [30]. Similarly pAKT has also been shown to be a critical regulator of cell survival and cell cycle progression apart from negatively regulating p53 [31]. Hence, we next checked the expression of p53, total AKT and pAKT in presence or absence of varied doses of Gatifloxacin. Figure 5A and 5B showed activation of p53 at both transcriptional and translational levels. We found Gatifloxacin treatment at 100 and 200 µg/ml dose increases p53 expression by 1.4–2 fold (p<0.01) transcriptionaly and 1.8–2.45 fold (p<0.01) translationally in MIA PaCa-2 cells and 1.75–2.5 fold (p<0.013) transcriptionaly and 1.4–2.0 fold translationally in Panc-1 cells after 48 h (Figure 5Ai, 5Bi) as compared to vehicle treated cells. We didn’t find any change in the p53 mRNA level/protein level in both cell lines at 400 µg/ml of Gatifloxacin (Figure 5B). Since we got maximum expression at 200 µg/ml, we did time-course experiment at 200 µg/ml of Gatifloxacin and observed that p53 expression started increasing within 8 h of Gatifloxacin treatment, transcriptionaly (p = 0.032) as well as translationaly (p = 0.022) in Panc-1 cells but there was delayed increase in p53 expression in MIA PaCa-2 cells which triggers off significantly only after 24 h transcriptionaly (p = 0.034) as well as translationaly (p = 0.048) and reached maximum by 40 h (p<0.01) and beyond in both cell lines (Figure 5Aii, 5Bii). As shown in Figure 5Bi, Gatifloxacin (100, 200, 400 µg/ml) treatment caused significant decrease in pAKT (Ser 473) levels in a dose dependent manner from 0.86–0.51 fold (p = 0.027) in MIA PaCa-2 and 0.89–0.41 fold (p<0.01) in Panc-1 cells respectively without any change in total AKT levels. Our time–course experiment showed that up to 16 hrs there was not much decrease in p-Akt (Ser 473) levels but after 16 h there was *significant* decrease of pAKT levels (p<0.01) in MIA PaCa-2 cells. In Panc-1 cells, up to 24 h there was no decrease in pAKT levels and decrease became significant from 32 h (p<0.01) onwards in the presence of 400 µg/ml of Gatifloxacin treatment (Figure 5Bii).

**Figure 6:**
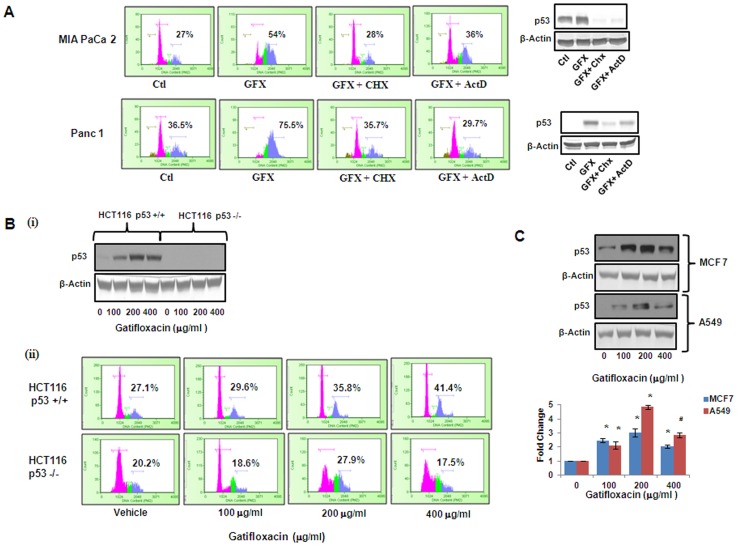
Gatifloxacin-induced G_2_-phase arrest is p53 dependent. (**A**) Left Panel shows the Cell cycle analysis of MIA PaCa-2 and Panc-1 cells when grown in the presence or absence of p53 transcriptional inhibitor ActinomycinD (1 µg/ml) or Translational inhibitor cycloheximide (2 µg/ml) along with 200 µg/ml Gatifloxacin for 48 h and right panel shows the p53 protein expression in MIA PaCa-2 and Panc-1 cells in presence or absence of 200 µg/ml Gatifloxacin with or without 2 µg/ml CHX or 1 µg/ml ActD. % here indicates percentage of G_2_ phase subpopulation**.** (**B**) (**i**) Western blot analysis for p53 **e**xpression in HCT116 p53+/+ and P53−/− cell lines treated with Gatifloxacin in a dose dependent manner for 24 h. (**ii**) Cell cycle analysis of HCT116 p53+/+ and P53−/− treated with Gatifloxacin (0–400 µg/ml) for 24 h. % here indicates percentage of G_2_ phase subpopulation. (**C**) Western blot analysis of p53 protein **e**xpression in MCF 7 and A549 cells treated with Gatifloxacin in a dose dependent manner. Bar Graph represents the mean ± SEM. *p<0.01, compared to control.

Next, in order to investigate whether the arrest induced by Gatifloxacin is p53 dependent, the transcription inhibitor ActinomycinD (ActD) and protein synthesis inhibitor Cycloheximide (CHX) was used and cell cycle analysis was performed. As shown in Figure 6A in the presence of 200 µg/ml of Gatifloxacin there was complete G_2_-phase arrest, which was abolished in presence of CHX (2 µg/ml) and ActD (1 µg/ml) after 48 h in both the cell lines. Right panel of the figure shows the efficacy of CHX and Act D in reducing p53 levels. Our results suggested that Gatifloxacin causes G_2_-phase arrest in a p53 dependent manner. We couldn’t use siRNA here because it is very well documented that both MIA PaCa-2 and Panc-1 have mutation in p53 [32]. To further confirm the role of p53 in Gatifloxacin induced G2 arrest, a comparison between the responses in isogenic HCT116 wild type p53+/+ and deficient p53−/− cell lines was done. We treated both the cell lines with Gatifloxacin (100, 200, 400 µg/ml) for 24 h and found significant increase in p53 protein level in wild type HCT116 p53+/+ which was similar to MIA PaCa-2 and Panc-1. This induction however, was not observed in p53 deficient HCT116 p53−/− cells treated under the same conditions (Figure 6Bi). We also compared the cell population arrested in the G2-phase after Gatifloxacin treatment of both cell lines HCT116 cell lines. Our data showed significant increase in G2-phase arrest from 27% to 41% in HCT p53+/+ cell line but no change in G2-phase subpopulation in HCT p53−/− cell line thereby pointing towards our theory of p53 as a target of Gatifloxacin (Figure 6Bii). We then also confirmed the effect by simultaneously checking the expression of p53 protein in a dose dependent manner (Gatifloxacin 0, 100, 200, 400 µg/ml) in two other p53 positive cell lines MCF7 (2–3.08 fold increase, p = 0.0078) and A549 (2.1–4.8 fold increase, p = 0.0085) respectively (Figure 6C).

### Gatifloxacin Decreases the Levels of S and G_2_–phase Regulatory CDKs and Cyclins in Both Cell Lines

**Figure 7:**
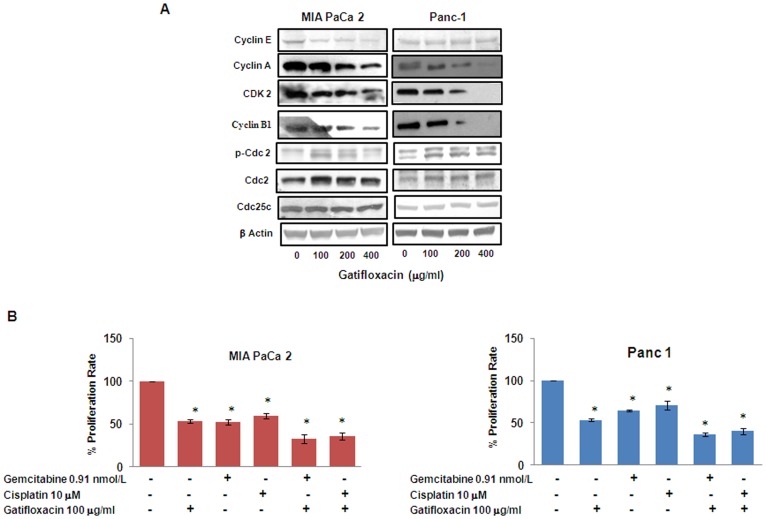
Gatifloxacin affects cell cycle regulatory proteins and synergizes the antiproliferative effects of other broad spectrum anticancer drugs. (A) Effect of Gatifloxacin on S and G_2_-phase regulatory Cyclins and CDKs as assessed by western blot analysis in MIA PaCa-2 and Panc-1 cells (B) Gatifloxacin (100 µg/ml) synergizes the antiproliferative effect of Gemcitabine (0.91nM/L) and Cisplatin (10 µM) in MIA PaCa-2 and Panc-1 cells as assessed by MTT assay. Vertical axis represents % proliferation rate whereas Horizontal axis represents presence or absence of above mentioned drugs. Data are mean ± SEM of three independent experiments performed in triplicate. *p<0.02 as compared to untreated control.

To dissect the biochemical events controlling the transition from one cell cycle phase we examined the levels of several cell cycle proteins following treatment with Gatifloxacin. Cyclin A, Cyclin E and CDK2 regulates normal S-phase progression [33]; [34] and we found Gatifloxacin to significantly decrease the expression levels of these proteins in both the cell lines in a dose dependent manner after 48 h (Figure 7A). In a similar manner Gatifloxacin treatment in both the cell lines also caused down regulation of Cyclin B1 expression, which is involved in G_2_-phase progression. However, we found increase in the expression of inhibitory phosphorylation of cdc2 at tyr-15 as well as total cdc-2 levels in both the cell lines, whereas there was no change in the expression of Cdc25c.

### Gatifloxacin Synergizes the Effect of Gemcitabine and Cisplatin in Pancreatic Cancer Cells

Gemcitabine (2′, 2′-difluoro-2′-deoxycytidine) is very well known and the most effective drug for pancreatic cancer so far and its combination with Cisplatin is found to be moderately effective against advanced stages of cancer [35]. There was only 26% response rate in Gemcitabine-Cisplatin mediated chemotherapy as compared to <10% in gemcitabine alone therapy and occurrence of resistance against both of them has aggravated the situation [36], [37]. We next tried to investigate whether Gatifloxacin has any effect on the inhibitory activity of Gemcitabine or Cisplatin (broad spectrum anti cancer drug) *in vitro*. As shown in Figure 7B Gemcitabine (0.91nM/L) alone was able to suppress the proliferation by 45±2.9% and 33±1.4% in MIA PaCa-2 cells and Panc-1 cells respectively which was enhanced to 66±5.1% (p = 0.016) and 64±2% (p = 0.011) after 48 h in presence of 100 µg/ml Gatifloxacin. Similarly Gatifloxacin was also found to synergize the antiproliferative activity of Cisplatin (10µM) from 42±3% to 66±1.5% (p = 0.018) in MIA PaCa-2 cells and from 30±5% to 61±3% (p = 0.024) in Panc-1 cells treated for 48 h.

## Discussion

Here in for the first time, we show convincingly that Gatifloxacin inhibits cell proliferation in pancreatic cells by arresting them in S and G_2_-phase in time and dose dependent manner without any induction of apoptosis. In conjunction to our findings, the antitumor activities of several other fluoroquinolones have been reported in several cancer cell types [38]–[41].

It is well known that eukaryotic cell cycle is regulated by the coordinated activity of protein kinase complexes, each consisting of a cyclin-dependent kinase (Cdk) and cyclins. Cdk kinase complexes are formed and activated at specific stages of the cell cycle, and their activities are required for progression through distinct cell cycle phases [42]. Progression through G1 and entry into S-phase is regulated by the Cyclin A–Cdk2 and Cyclin E–Cdk2 complexes, respectively, and the G2/M phase transition is driven by Cyclin B–Cdc2 [43]. It is also known that these Cyclin–Cdk complexes often bind to the endogenous inhibitor proteins (CKIs) p21WAF1/CIP1 and p27KIP1, which inhibit their kinase activities and prevent cell cycle progression. Consistent with this notion in the present study, we demonstrate that Gatifloxacin inhibits Cyclin A, cyclin E, Cdk2 and cyclin B1 expression. In addition to these we observed increased inhibitory phosphorylation (Tyr^15^) of cdc2 and no change in cdc25c expression levels following gatifloxacin treatment in both the cell lines. In our study we have also observed increase in the protein levels of inhibitory p21 (in MIA PaCa-2) and p27 (in Panc-1) cells respectively. It seems that increased phopshorylation of cdc2 on Tyr^15^ and increased p21/p27 levels could be possible explanation by which pancreatic cancer cells undergo cell cycle arrest during Gatifloxacin exposure. However additional experiments will be needed to provide a conclusive answer.

Herein, we further show that Gatifloxacin causes TGF-β1/p21 dependent S-phase arrest specifically in MIA PaCa-2 cells and p27 dependent S-phase arrest in Panc-1. Our study is in accordance to a report of Bourikas *et al* where they have shown that ciprofloxacin exerts its antiproliferative effects on colon cancer cells by arresting them in S-phase in a TGF-β1 dependent manner. It is well documented that TGF-β signaling pathway plays a pivotal role in pancreas development and functions at embryonic as well as at adult levels [44], whereas loss of TGF-β signaling leads to autoimmune pancreatitis and pancreatic cancer [45]. It is also known that pancreatic cancer expresses low levels of TGF-βR1 which ultimately lead to non responsiveness towards TGF-β1 [46]. Literature shows that TGF-β conveys its growth inhibitory signal from receptor to the nucleus through downstream signaling proteins SMAD. TGF-β directly activates and phosphorylates R-SMADS (Receptor regulated SMADS: Smad2, Smad3) which ultimately forms heteromeric complex with Smad4 and translocates to the nucleus and these Smad complexes then alter the transcriptional response or growth inhibition usually by activating Cyclin dependent kinase inhibitor p21^Waf1/Cip1^ [47] Consistent to these findings in the current study we observed activation of Smad-3 (but not Smad-2) and its translocation to the nucleus after binding to Smad4 and thereby activating p21^Waf1``/Cip1^.

Apart from being regulated by TGF-β1, p21 is also known to be tightly controlled by the tumor suppressor protein p53, through which this protein mediates the p53-dependent cell cycle arrest in response to a variety of stress stimuli [30]. Literature also shows that p53 mediated G_1_ and G_2_ cell cycle arrest is accomplished through transcriptional activation of its downstream targets p21, GADD45α, 14-3-3ε [48]. In our study we used MIA PaCa-2 and Panc-1 which are mutant p53 cell lines and found that Gatifloxacin was able to activate p53 upto 200 µg/ml, but we did not find its activation at 400 µg/ml in both the cell lines. Effect of Gatifloxacin on p53 and G_2_ arrest was reversed when blocked with ActD and CHX, which shows p53 dependency transcriptionally as well as translationally. Surprisingly in our study activated p53 was unable to activate GADD45α, 14-3-3ε (data not shown) and p21 being regulated by TGF-β1, but still it was able to cause G_2_-phase cell cycle arrest in both cell lines. Activation of p53 suggested that the p53 pathway also plays a crucial role in Gatifloxacin induced G_2_-phase cell cycle arrest in both the cell lines, which was further validated in wild type p53+/+ and null p53−/− HCT116 cell lines. These results were also confirmed in other wild type cell lines A549 and MCF7.

**Figure 8:**
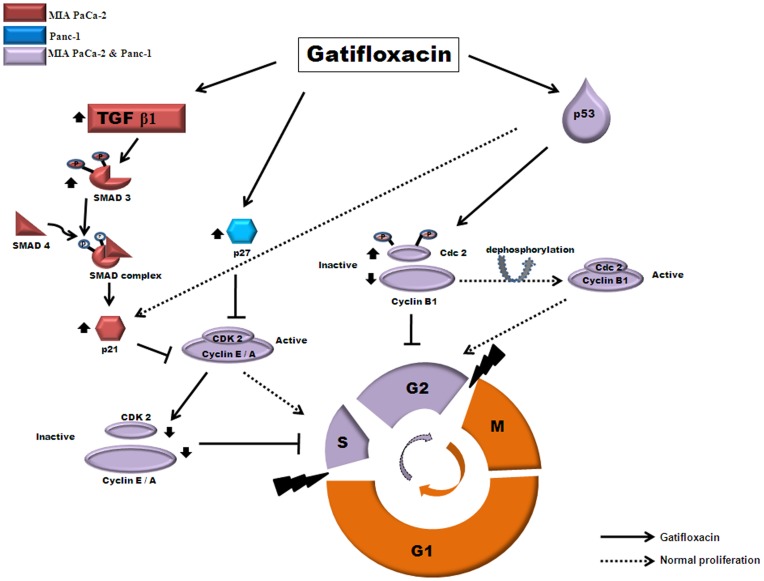
Schematic representation of proposed mechanism of action of Gatifloxacin induced S and G_2_ phase arrest in pancreatic cancer cells. Gatifloxacin leads to activation of TGF-β1 only in MIA PaCa-2 cell line which than activates Smad-3 but not Smad-2 via its phosphorylation and forms complex with Smad-4 in cytoplasm. Complex than translocates to the nucleus where it activates p21, however in case of Panc-1 Gatifloxacin directly activates p27. p21 (waf1/cip1) and p27 (kip1) both being cyclin dependent kinase inhibitor, than inhibits and down regulates Cyclin A, Cyclin E and CDK-2 which than causes S-phase arrest in both the cell lines. Gatifloxacin also activates p53 in both MIA PaCa-2 and Panc-1 cell lines which than down regulates Cyclin B1, upregulates inhibitory pCdc2 and Cdc2 which than leads to G_2_ arrest. However, activation of p53 doesn’t affect the levels of Cdc25c. pAKT, an inhibitor of Smad complex, p21, p27 and p53 was found to be downregulated in both cell lines under the effect of Gatifloxacin.

Literature suggests that AKT (serine/threonine kinase), is responsible for providing resistance to chemotherapy as it is found to be hyperactive in pancreatic cancer and reports have shown that inhibition of AKT signaling increases the chemosensitisation of drugs [49]. Our data clearly shows that Gatifloxacin not only downregulates AKT but also synergizes the effect of Gemcitabine and Cisplatin in Pancreatic Cancer cells. According to above results it was tempting to speculate a model for the action of Gatifloxacin in pancreatic cancer cells as shown in Figure 8.

In conclusion, we have elucidated a new mechanism of action of Gatifloxacin against pancreatic cancer that is through induction of TGF-β1, p21, p27 and p53. The fact that Gatifloxacin synergizes the effect of anticancer drugs opens new insight into the therapeutic index in pancreatic cancer treatment. However, future *in vivo* studies would help to determine therapeutic efficacy, is a matter that needs further investigation.

## Materials and Methods

### Reagents and Antibodies

DMEM, Antibiotic Antimycotic solution, Trypsin EDTA, DMSO, propidium iodide, protease and phosphatase inhibitor cocktail, BCIP-NBT, BCA reagent, Cisplatin, Cycloheximide, Actinomycin D, Pre designed TGFβ1 siRNA and scrambled siRNA were purchased from Sigma (St. Louis, Missouri, USA). SMART pools of siRNA against p21 and p27 were purchased from Dharmacon RNAi technologies (Denver, CO, USA). MTT was purchased from Amresco (Ohio, USA). Fetal bovine serum was purchased from Biological Industries (Kibbutz Beit Haemek, Israel). Human recombinant TGF-β1 and antibodies Cyclin A, Cyclin E, Cyclin B1, CDK-2, p21, p27, Skp2 was purchased from Cell signaling technologies (MA, USA). Antibodies pSmad-2, pSmad-3, Smad-2, Smad-3, Smad-4, TGF-β1, α-Tubulin and secondary antibodies were from AbCam (Cambridge, MA, USA). β-Actin, pAKT (Ser 473), AKT, Bax, p53, pCDC-2 (Tyr-15), CDC-2, CDC25c and Lamin-B antibodies were purchased from Santacruz biotechnology (Santa Cruz, CA, USA). Caspase 3,8,9 activity kit was purchased from G-Biosciences (St Louis, MO, USA). Gatifloxacin was obtained from Cipla (India) and Gemcitabine was obtained from Eli Lily (India).

### Cell Culture and Transfection

MIA PaCa-2 and Panc-1 cells were obtained from National Center for Cell Science, Pune, India and maintained in DMEM medium containing 10%(v/v) FBS, 100 units/ml penicillin, 100 µg/mlstreptomycin, 0.25 µg/ml, amphotericin B in a humidified 5% CO_2_ atmosphere. Cells growing in logarithmic phase were used in all experiments. Overnight serum starved cells were used to study TGF -β1, p53 and pAKT expression. Synchronized and growth arrested cultures were than subjected to Gatifloxacin (0–400 µg/ml) treatment in complete media for 24h and 48h respectively. Wherever indicated siRNA transfections were done in 6 well plate using lipofectamine 2000 (Invitrogen, CA, USA) according to manufacturer’s protocol. Cells were treated with Gatifloxacin, 24 h post transfection and were harvested after trypsinization for cell cycle experiment.

### Cell Viability Assay

Cell viability assay was performed using MTT [3-(4,5-dimethyl thiazol-2yl)-2,5-diphenyltetrazolium bromide]. 10,000 cells per well were seeded in 96 well plates and treated with different concentrations (0–400 µg/ml) of Gatifloxacin in triplicates. As controls, Dextrose 5% (w/v) treated cells (Vehicle) were included in each experiments. Following treatments for 24h and 48 h, 10 µL of MTT (5 mg/ml) was added to each well and incubated for 3 h at 37°C in dark. Formazan crystals formed were dissolved in 100µl DMSO and the absorbance was measured at 570 nM using an ELISA reader. Cell viability was calculated as reported earlier [50].

### Flow Cytometric Analysis and Caspase Activity for Apoptosis and Cell Cycle Analysis

Apoptosis was assessed using Guava Nexin kit and Guava PCA system according to the manufacturer’s protocol (Guava Technologies, Hayward, California, USA). Annexin-PE fluorescence was analyzed by cytosoft software (Guava Technologies, Hayward. California, USA). For analysis of cell cycle distribution, 1×10 ^6^ cells were harvested by centrifugation, washed in phosphate-buffer saline (PBS), fixed with ice cold 70% ethanol and treated with 1 mg/ml RNAse for 30 min. Intracellular DNA was labeled with propidium iodide (50 µg/ml) and incubated at 4°C in dark. Samples were than analyzed using flow cytometer (Guava Technologies, Hayward, California, USA) and cytosoft software (Guava Technologies, Hayward, California, USA). A minimum of 5,000 events were counted [51]. Caspase activity was measured using G-Biosciences Caspase 3, 8, 9 kit according to manufacturer’s protocol.

### Real Time PCR

Total RNA was extracted using Trizol reagent (Invitrogen, CA, USA). Reverse transcription was carried out using M-MuLV reverse trancriptase (MBI Fermentas, USA) according to the manufacturer’s protocol using 1 µg of total RNA. Real Time PCR for TGF-β1 and p53 was performed using SYBR Green PCR master mix (Applied Biosystems, Foster city, CA, USA) in an ABI Prism 7000 sequence detection system (Applied Biosystems), and amplification were performed in triplicate and repeated thrice. Results were normalized with 18S rRNA and analysis was done using pfaffl’s method [52]. Primers sequences used for real time PCR were as follows: **TGF-β1** FP: **5′**GCCCTGGACACCAACTATTG**3’** RP: **5′**CGTGTCCAGGCTCCAAATG**3’, p53** FP: **5′**TTGCAATAGGTGTGCGTCAGA**3’** RP**: 5′**AGTGCAGGCCAACTTGTTCAG**3’** and **18S** FP: **5′**GTAACCCGTTGAACCCCATT**3’** RP: **5′**CCATCCAATCGGTAGTAGTAGCG**3’**.

### Preparation of Cell Lysates and Immunoblot Analysis

Cell pellets obtained after treatment with Gatifloxacin (0–400 µg/ml) were lysed with cell lytic buffer containing protease/phosphatase inhibitor cocktail purchased from Sigma (St. Louis, Missouri, USA). Protein concentration was determined using BCA (Sigma, StLouis, Missouri, USA) protein estimation kit. Equal amount of sample lysate (90 µg for p21, p27 and 50 µg for rest of the proteins) were separated by SDS-PAGE and transferred to PVDF membrane. The membrane was blocked with 5% skim milk (3% BSA in case of phospho-protein) in TBST and probed with primary antibody overnight followed by incubation with appropriate secondary antibody (ALP or HRP linked). After washing, blots were developed using enzyme based chemiluminescence assays (alkaline phosphatase) by BCIP-NBT (Sigma, Missouri, USA) or enhanced chemiluminescence ECL western blot detection system (Pierce, Illinois, USA). Measurement of signal intensity of protein expression on PVDF membrane was done using alphaimager 3400 (Alpha Innotech Corporation, San Leandro, California, USA) and normalized using β-Actin as whole cell loading control and Lamin as nuclear control [53]. All data were expressed as fold change. All the experiments were repeated three times; representative results are presented.

### Subcellular Fractionation

Gatifloxacin treated MIA PaCa-2 cells were washed twice with PBS, pelleted at 5000 rpm for 10 min. The Pellets were resuspended by gentle pipetting in 100 µl of ice-cold Cytoplasmic lysis buffer (Pierce, Illinois, USA) containing protease inhibitor and was subjected to vigorous vortexing for 10 sec followed by incubation in ice for 1 min. Cycle was repeated 5 times and supernatant was isolated by centrifugation at 7500 rpm for 5 min, remaining pellet was resuspended in 20 µl of Nuclear lysis buffer (Pierce, Illinois, USA) and vortexed vigorously at 4°C for 30 min. The suspension was centrifuged at 12,000 rpm for 20 min. α-Tubulin and LaminB was used to check the purity of Cytoplasmic and Nuclear fractions respectively.

### Statistical Analysis

Results are given as mean of three independent experiments ± SEM. Statistical analysis was performed with student’s two tailed t-test using SPSS (windows version 7.5); values of p≤0.05 were considered statistically significant.
